# An iron-based molecular cathode for alkaline electrocatalytic hydrogen evolution

**DOI:** 10.1039/d5ra09721e

**Published:** 2026-04-20

**Authors:** Bharath M, Srijit Sen, Bhupendra P. Mali, Munmun Ghosh

**Affiliations:** a Department of Chemistry, Ashoka University Sonipat Haryana-131029 India munmun.ghosh@ashoka.edu.in

## Abstract

Herein, we investigate the heterogenization of a molecular iron complex containing a *p*-chloropyridine moiety through simple electroanalytical techniques and explore its electrocatalytic HER efficacy. The modified electrodes are active in alkaline aqueous solutions, have low overpotentials (0.35 V *vs.* RHE) and showed stability for over 150 hours under electrolysis conditions.

## Abstract 2

This article discusses a simple and reliable electrochemical methodology for heterogenizing iron-based molecular catalysts on conducting carbon surfaces.

## Graphical abstract

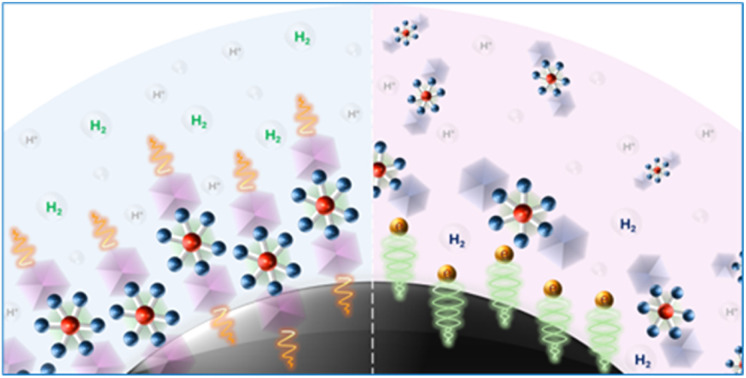

## Introduction

Transition metal complexes have become a significant class of electro-catalysts for reductive and oxidative electrochemical processes due to their adaptable structural characteristics and their ability to have a variety of oxidation states. Recently, a large and still-growing body of work has been dedicated to combining metal–ligand cooperativity with redox-active ligands. In many cases, the flexibility of molecular engineering is utilized to enhance the catalytic efficiency and stability of the active centres by adjusting the electronic effect from the inner and outer coordination sphere of the catalyst.^1^

Recent achievements made with polypyridyl and substituted pyridyl complexes show that synergy between metals and redox-active ligands results in reliable and efficient catalytic activity. Representative examples of this family of catalysts are the 3d transition metal complexes of pyridine-2,6-disubstituted ligands.^2^ Their versatile reactivity is attributed to the stability offered by the pincer cavity created by the five-membered chelate rings. The modularity of molecular engineering offered by these complexes tunes the electronic properties as well as the reactivity of these complexes. Recently, several groups, including ours, have shown that the presence of electron-donating groups in pyridine-2,6-disubstituted complexes improves their electrocatalytic properties for the HER and the related carbon dioxide reduction reaction.^3,4^ However, anchoring of these complexes for direct aqueous electrocatalytic applications has not yet been explored. This inspired us to analyse the potential of these catalysts as a heterogenized electrode material for the water-splitting reaction. For this, we chose bis-chelated Fe(iii) molecular anionic complexes bearing a chloro-substituted pyridine-2,6-dicarboxylate ligand. Heterogeneous catalysts are commonly loaded on electrode supports using binders. However, the presence of binders affects the activity, stability and durability of the electrodes at high current densities.^5^ Thus, alternative approaches such as the immobilization of molecular catalyst on conductive supports have emerged as an effective strategy; in most cases, this approach involves a structural modification in the ligand architecture.^6^ Some key examples include the immobilization of a nickel bisdiphosphine ([Ni(P_2_N_2_)_2_]^2+^)-based catalyst through covalent (amide coupling, diazonium reduction) and noncovalent immobilization (π–π stacking interactions through the inclusion of pyrene substituents) by Artero and coworkers,^7^ the immobilization of [Cp*Rh(L)Cl]^+^*via* pyrene functionalization by Brunschwig, Roberts and coworkers,^8^ and the non-covalent immobilization of cobalt corrole complexes *via* π–π interactions by Cao and coworkers,^9^ among others.^10,11^ In all of these cases, the modified electrodes showed excellent catalytic hydrogen production. However, these systems are unsuitable for implementation at an industrial scale due to their lack of stability, high cost and challenges in their synthesis.^6^ An ideal solution to this would be a catalyst that could be obtained with minimal synthetic effort using inexpensive materials and directly immobilized without further modification.^12^ To achieve this goal, we herein report an iron complex that is afforded in high yield from a one-pot reaction with inexpensive materials and can be immobilized electrochemically on a carbon surface *via* electro-oxidation reactions. Detailed structural and surface characterization of CFP electrodes functionalized with complex 1 (1-CFP) confirmed the immobilization. The modified electrode 1-CFP was further used as a working electrode for electrochemical HER in an aqueous KOH medium. The 1-CFP electrodes showed excellent electrochemical stability under electrolysis conditions for 150 hours with an overpotential of *η* = 350 mV at a current density of 10 mA cm^−2^. It is noteworthy that the immobilization of this catalyst necessitates no synthetic alteration, making it optimal for large-scale applications, unlike other catalyst designs that require structural adjustments.

## Results and discussion

### Synthesis and characterization

**Fig. 1:**
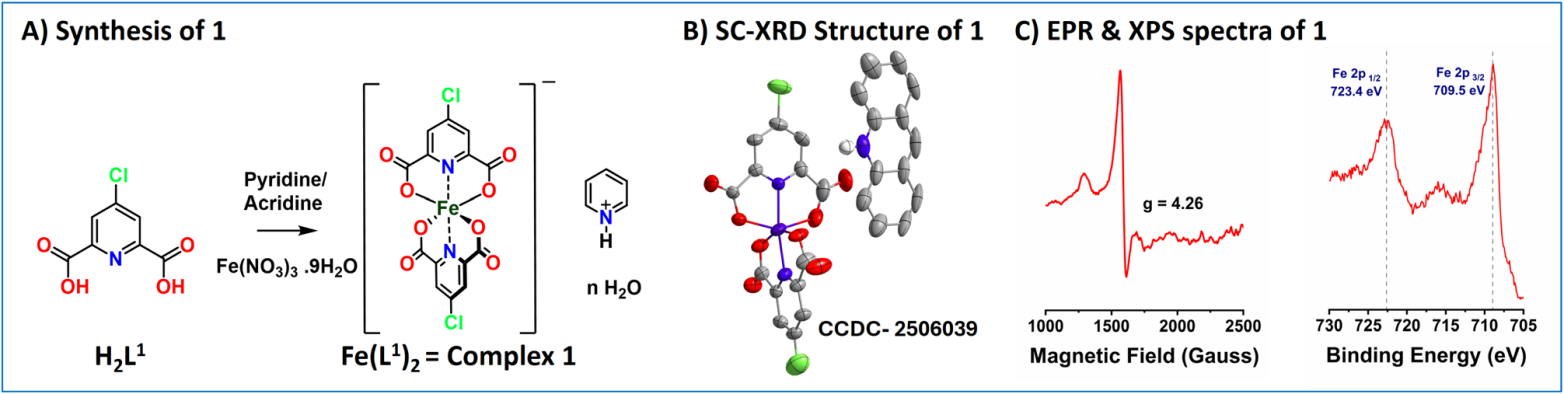
(A) Synthesis of Fe(L^1^)_2_ (1, with counter cation pyridinium ion). (B) Single crystal X-ray diffraction (SC-XRD) structure of complex 1 (with counter cation acridinium ion; H atoms have been omitted for clarity). (C) X-band EPR spectra of complex 1 at 77 K (*v* = 9.631 GHz, modulation frequency = 100 kHz, modulation amplitude = 10 G) and XPS spectra of complex 1 for the element Fe.

The ligand H_2_L^1^ was prepared by following a reported literature procedure with necessary modifications (Scheme S1 and Fig. S1–S2).^13^ The synthesis of 1 involved the aerobic reaction of the ligand H_2_L^1^ and pyridine/acridine with Fe(NO_3_)_3_·9H_2_O in a methanol/water mixture at 373 K (Scheme S2). Slow evaporation of the solvent from this solution afforded yellow blocks of 1 (when pyridinium was the counter cation, needle-like crystals were formed, and when acridinium was the counter cation, small block crystals were formed). The structural assignment of complex 1 was verified *via* single-crystal X-ray diffraction (SC-XRD) and electron-spray ionization mass spectrometry (ESI-MS), and the purity of the samples was verified using elemental analysis. Complex 1 crystallizes in a triclinic crystal system with the *P*1̄ space group (Fig. S3). As shown in Fig. 1A and B, the iron atom is coordinated by two deprotonated ligands in a meridional geometry, with coordination by four deprotonated carboxylate oxygen atoms in the equatorial plane and two pyridyl nitrogen atoms at the axial positions. The ESI-MS of complex 1 revealed a molecular ion peak at *m*/*z* 453.8695 (C_14_H_4_Cl_2_FeN_2_O_8_), which corresponds to a hexa-coordinated bis chelated system (Fig. S4). The X-band EPR spectrum of 1 in DMF at 77 K showed a signal with a *g* value of 4.26, corresponding to a high-spin (HS) Fe(iii) centre (Fig. 1C).^14^ Furthermore, XPS analysis of these complexes showed two peaks at 723.4 and 709.5 eV, indicating the presence of a Fe(iii) centre (Fig. 1c and S5).^3^

### Electrochemical immobilization and characterization of modified electrodes

In an attempt to immobilize complex 1 onto conductive carbon surfaces, we conducted electrochemical scanning of 0.1 mM catalyst solutions (0.1 M TBAF/DMF) at positive potentials (0 to 1.2 V) at a scan rate of 100 mV s^−1^ on carbon fibre paper (CFP) electrodes (see SI for control studies, Fig. S6–S8).^15^ Oxidation events were observed in the case of complex 1 at 0.55 V and 0.85 V, and on successive cycles (5 cycles) the current response for this event was significantly reduced, indicating masking of the active surface area of the working electrode.^16^ CV analysis of the catalyst-loaded carbon surfaces reveals that compound 1 is bound to the surface, showing quasi-reversible redox couples at potentials comparable to those of complex 1 in solution (Fig. S9–S11).^6^ The immobilization of complex 1 involves the anodic oxidation of chlorine to generate electrophilic halogen species and 1˙.^17^ When similar experiments were performed with control catalyst 2 (H_2_L^2^ = picolinic acid) and 3 (H_2_L^3^ = chelidamic acid), no such oxidation events or traces of electrochemical deposition were noted (Fig. S6). This strengthens the idea of electrochemical immobilization through homolytic C–Cl bond cleavage.^18^ As chlorine is a better leaving group, only complex 1 underwent electrochemical immobilization (see SI for more information, Scheme S3). The radical pathway followed for electrodeposition for complex 1 was further confirmed through EPR experiments (Fig. S7). For these, electrochemical deposition reactions were conducted in the presence of 5,5-dimethyl-1-pyrroline *N*-oxide (DMPO) under inert conditions using dry solvents, and aliquots of the reaction mixture collected during the electrodeposition reaction were characterized through EPR analysis. A quartet with unequal splitting was visible in the EPR spectra.^15,16,19,20^ A recent study by Wang and coworkers validated the utilization of DMPO as an effective strategy to scavenge Cl˙.^20^ Unlike the DMPO-OH adduct, a weak/broad EPR spectra is observed for the DMPO-Cl adduct with unequal splitting. Dry solvent conditions and high DMPO concentrations are necessary to trap the DMPO-Cl adduct, as Cl˙ is highly reactive and rapidly undergoes secondary reactions with water. Radical scavenging experiments verified the homolytic C–Cl bond cleavage and Cl˙ formation during the electrodeposition.^20,21^

To confirm the reliability of electrodeposition, several 1-CFP electrodes were prepared under identical conditions and tested. Electrochemical investigation of these electrodes yielded identical results, ensuring the reliability of the electrodeposition technique. Before performing any structural or surface characterization, the 1-CFP electrodes were washed with DMF to confirm the immobilization of 1 on the CFP surface. The nature of the electrodeposited species was analysed *via* solid-state FTIR of the 1-CFP electrode. Electrodeposited species were peeled from the electrode surface *via* physical means for this purpose.

The FTIR spectra of complexes 1, 2 and 3 displayed characteristic C=O, C–O, and C–N stretching bands at 1658, 1243 and 1341 cm^−1^, respectively (Fig. S12). In the FTIR analysis of bare CFP electrode, no characteristic bands were observed in the range of 600 cm^−1^ to 2000 cm^−1^ for bare CFP. The electrodeposited species were further evaluated using UV-visible and dynamic light scattering experiments. For these, several 1-CFP electrodes were dispersed in DMF solution and sonicated for a few hours. When the resultant solutions were examined, their UV-vis spectra showed characteristic π–π* and LMCT transitions similar to those of complex 2 (Fig. S13). Notably, the DLS experiment verified the absence of any nanoparticle formation (Fig. S14). Collectively, these experiments verified the molecular nature of the electrodeposited species.

**Fig. 2:**
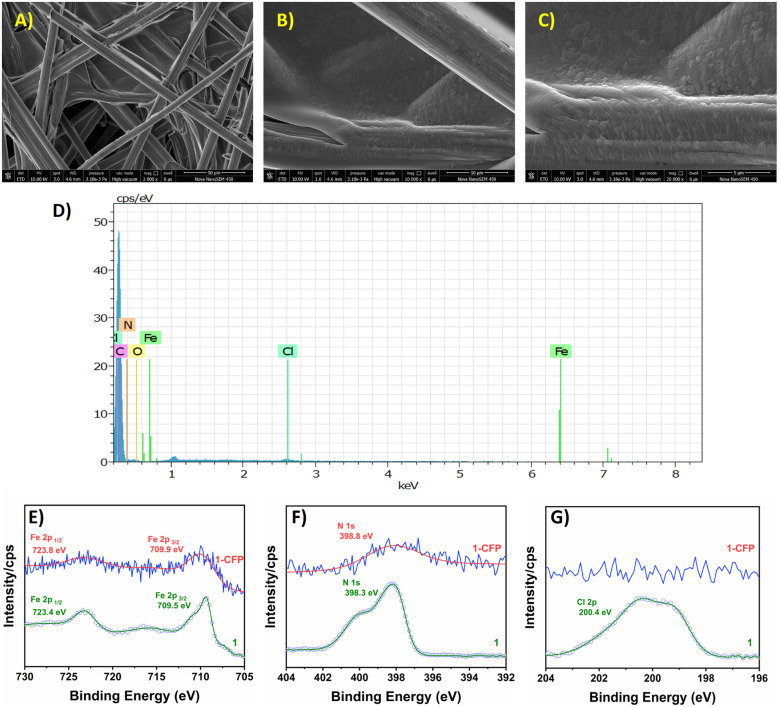
FESEM images of modified electrode 1-CFP with different magnifications: (A) 50 µm, (B) 10 µm and (C) 5 µm. (D) EDS spectra of 1-CFP. Core level XPS spectra of 1-CFP (E) Fe 2p, (F) Cl and (G) N 1s. No peaks were observed in the region of 190 to 210 eV, suggesting the absence of Cl atoms in the modified electrode.

The elemental composition of the electrodeposited molecular species was further confirmed from FESEM-EDX and XPS analysis of the modified electrode 1-CFP (Fig. 2).^12^ Morphology analysis using FESEM revealed that an undefined surface resembling a flaky scale was formed on the carbon fibre due to the deposition of complex 1 (Fig. S15). Moreover, FESEM-EDX elemental mapping highlighted the presence of the elements Fe, C, N and O on the 1-CFP electrode. Notably, the element chlorine was either completely absent or nearly absent in the elemental mapping; XPS analysis of 1-CFP electrodes produced similar findings. Fig. S16 shows the survey scan spectrum of 1-CFP, which confirms the presence of the major constituent elements Fe, C, N and O in 1-CFP. In Fig. 2E, the peaks at 723.8 and 710.8 eV are assigned to the Fe 2p_3/2_ and Fe 2p_1/2_ electronic states of iron in the +3 oxidation state as present in the molecular complex before electrodeposition. The N 1s spectrum of 1-CFP displays a peak at 398.3 eV corresponding to the N atom, as shown in Fig. 2F. Interestingly, no noticeable peaks were observed in the region between 190 to 210 eV, suggesting a minimal amount or no chlorine was present in the modified electrodes (Fig. 2G and S17). Thus, both XPS analysis and FESEM-EDX analysis indicated the absence of the element chlorine in the electrodeposited species. This observation is in good agreement with the mechanism proposed for the electrodeposition of complex 1.

### Electrocatalytic hydrogen evolution activity

**Fig. 3:**
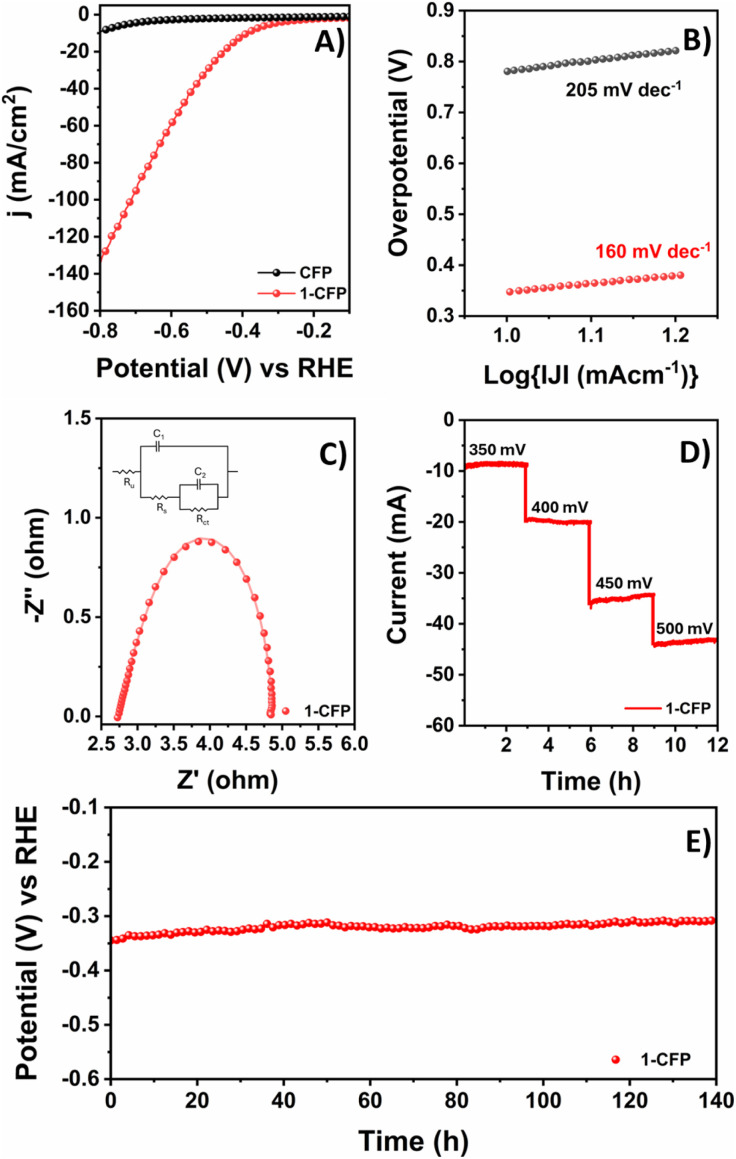
(A) Linear sweep voltammogram of 1-CFP (red trace) and bare CFP (black trace). (B) Tafel slope of 1-CFP and CFP in the HER. (C) Electrochemical impedance spectra obtained at an overpotential of 300 mV for the 1-CFP electrode (inset: electrical equivalent circuits (*R*_ct_) used for the fitting of the experimental data and calculation of parameters). (D) Potential-dependent chronoamperometry stability test of 1-CFP. (E) Chronopotentiometry stability analysis of 1-CFP.

To investigate the electrocatalytic activity of 1-CFP, electrochemical measurements were carried out in 1 M KOH (pH = 13.95) solution (Fig. 3A). The quantitative HER performance of the modified electrodes was evaluated using linear sweep voltammetry (LSV) experiments. Notably, 1-CFP showed a rapid increase in current near −0.4 V *vs.* RHE, unlike the flat response shown by the bare CFP electrode. Reproducibility tests of the electrodes were conducted with identical electrodes under identical conditions, and the results confirmed the catalytic activity of the modified electrode (Fig. S18).

We then replaced the KOH solution (after electrochemical analysis) with a new electrolyte solution, and the recorded polarization curves showed similar results to those observed earlier. These results indicate that the catalyst-deposited CFP electrodes are stable on the surface and resistant to leaching under electrochemical conditions. The HER reaction kinetics were evaluated through Tafel analysis for the 1-CFP electrodes and showed a Tafel slope value of 160 mV dec^−1^. The comparatively lower Tafel slope compared to that of the bare CFP electrode (205 mV dec^−1^) indicated faster electron transfer kinetics and better HER performance (Fig. 3B).^22^ Additionally, the reaction kinetics were evaluated using electrochemical impedance spectroscopy (EIS). Impedance studies recorded at frequencies from 100 kHz to 0.1 Hz at a potential of −1.5 V revealed a semi-circular feature for both CFP and 1-CFP (Fig. 3C). The smallest semi-circle was recorded for the 1-CFP electrode, indicating better charge conduction at the electrode–electrolyte interface, which facilitated better HER performance (Fig. S19).^23^

Electrochemically active surface area (ECSA) calculations also supported the increased activity of 1-CFP. These results indicated that the 1-CFP surface is intrinsically more active towards the HER due to the immobilization of complex 1. The better electron transfer kinetics after catalyst immobilization ensured good conductivity, while the porous structure provided a larger surface area; collectively, these factors boosted the electrocatalytic performance of the modified electrode. After verifying the enhanced electrocatalytic activity of 1-CFP, we were interested in analysing the electrochemical stability of the electrodes under electrolysis conditions (Fig. 3D and E). A consistent current density was observed in chrono-amperometry studies performed at different applied overpotentials for 3.0-hours intervals for the 1-CFP electrodes. Analogous chronopotentiometry experiments performed at 10 mA cm^−2^ also demonstrated enhanced durability. During sustained reaction at constant current densities, nearly constant voltage responses were obtained for the 1-CFP electrodes. GC-TCD analysis of the gas collected from the headspace of the bulk electrolysis cell at regular intervals showed that hydrogen was produced as the major product with a faradaic efficiency of 94%. The quantitative faradaic efficiency of 1-CFP electrodes suggests its stability under electrochemical conditions (Fig. S20). Interestingly, comparison of the polarization curves for the HER before and after the bulk electrolysis reaction demonstrates identical responses. As no significant loss in catalytic activity was observed in the voltammograms recorded before and after electrolysis, it was assumed that no structural change in the catalyst loaded on the carbon support occurs during the harsh electrolysis reactions (Fig. S21), unlike in previous reports in the literature.^24,25^

**Fig. 4:**
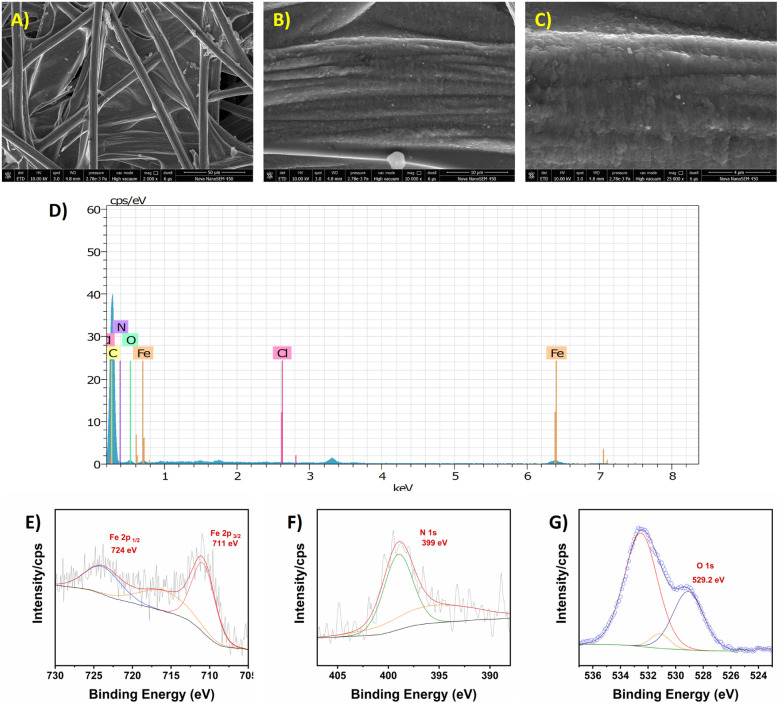
FESEM images of modified electrode 1-CFP after bulk electrolysis at different magnifications: (A) 50 µm, (B) 10 µm and (C) 5 µm. (D) EDS spectra of 1-CFP after bulk electrolysis. High-resolution XPS spectra of 1-CFP after bulk electrolysis: (E) Fe 2p, (F) Cl and (G) N 1s. No peaks were observed in the region of 190 to 210 eV, suggesting the absence of Cl atoms in the modified electrode.

To further confirm this, electrodes were characterized through FESEM-EDX and XPS analysis after bulk electrolysis (Fig. 4). Surface characterization of the 1-CFP electrodes after electrolysis experiments revealed that the original structure was retained. A flaky scale-like morphology was observed on the strands of the electrode after the electrolysis experiment (Fig. S22). FESEM-EDX elemental mapping highlighted the presence of the elements Fe, C, N and O on the 1-CFP electrode, confirming the presence of catalyst 1 on CFP. Further, XPS analysis was performed on these electrodes to confirm the structural integrity of the catalyst after electrolysis. The high-resolution spectra for the elements Fe, N, O and Cl showed similar results for 1-CFP electrodes before and after electrolysis. In Fig. 4E, the peaks at 724 and 711 eV are assigned to the Fe 2p_3/2_ and Fe 2p_1/2_ electronic states of iron in the +3 oxidation state, which is present in the molecular complex prior to electrodeposition and after electrodeposition. The N 1s spectrum of 1-CFP displayed a shoulder peak at 399 eV, and two O 1s peaks were observed at 527.7 and 531 eV, corresponding to the N and O atoms present in the modified electrode. As observed earlier, no noticeable peaks were observed in the region between 190 and 210 eV, indicating the absence of the element chlorine. Taken together, the electrochemical results and surface characterization of the electrodes support the stability of the 1-CFP electrodes.

## Experimental

### Materials and methods

Chemicals of high purity grade were purchased and used without further purifications (unless otherwise mentioned). Solvents, including methanol, dimethyl formamide, ethyl acetate, and chloroform, were purchased from Sigma Aldrich and were degassed *via* extended nitrogen purges prior to their use. For both synthesis and electrochemical studies, Milli-Q water (resistance: 18.2 MΩ cm) was used.

^1^H NMR spectra were recorded with a 400 MHz Bruker NMR spectrometer at room temperature using DMSO-*d*_6_ as the solvent. Chemical shifts (*δ*) are expressed in ppm, and the residual proton resonance of the solvent was used as an internal standard (DMSO: 2.5 ppm). Single-crystal data were collected using an Oxford XCalibur CCD diffractometer equipped with graphite monochromatic Mo-Kα radiation (*λ* = 0.71073 Å). Data reduction was performed with the CrysAllisPro program (Oxford Diffraction ver. 171.34.40). The structures were solved by direct methods using the SHELXL program and refined on *F*^2^ using all data by full-matrix least-squares procedures with SHELXL-2018/3. The hydrogen atoms were placed at calculated positions and included in the last cycles of the refinement. For complex 1, some disordered electron density could not be resolved, and therefore, the solvent masking procedure (PLATON SQUEEZE) was used.^26,27^ The crystallographic data collection and structure solution parameters are summarized in Table S1 (ESI). ESI mass spectroscopy was performed using an Agilent 6540 mass spectrometer. X-ray photoelectron spectroscopy (XPS) was performed using a Thermo Fisher Scientific X-ray photoelectron spectroscope with microfocused X-ray (400 µm, spot size: 72 W, 12 000 V). All spectra were collected using a monochromatic Al Kα source (*hν* = 1486.6 eV) and a hemispherical analyzer with 128-channel plate detectors. Charge compensation was achieved with a system flood gun that provided low-energy electrons and argon ions and reduced the charge shift. EPR spectra were measured using a Bruker A300-9.5/12/S/W spectrometer. The binding energies of all spectra were corrected using the difference between the observed C 1s peak energy and the peak energy of adventitious carbon (284.5 eV).^28^ Spectra were fit with a Shirley-type background using CASAXPS version 2.3.19PR1.0 software. EPR spectra of 1 were characterized at 144 K in dry dimethylformamide. 5 mM catalyst solutions were prepared in DMF, and the EPR tubes containing the catalyst solutions were dipped in liquid nitrogen for half an hour prior to data collection. FESEM images and energy-dispersive X-ray spectroscopy (EDX) data were collected with a JEOL scanning electron microscope equipped with an X-ray elemental analyzer. Transmission electron microscopy (TEM) analysis was performed using a JEOL JEM-ARM200F NEOARM. The absorption of light in the wavelength range of 300 to 800 nm was studied using UV-visible diffuse reflectance spectroscopy employing Cary series UV-vis (Agilent technology), and infrared spectra were recorded using an Agilent Cary 630 spectrometer with a diamond ATR unit at room temperature.

Working electrodes were prepared by electrodepositing complex 1 on carbon fiber paper. For this purpose, carbon fiber sheets were cut into rectangular pieces with dimensions of 1 cm × 2 cm. Prior to electrodeposition, the CFP electrodes were washed with DI water followed by ethanol and dried at 60 °C. The electrodes were then clamped to a soft electrode holder purchased from Dtech solutions (Gold-coated copper rod soft electrode holder, MTXH01) and used as working electrodes.

## Conclusions

In summary, an easily accessible ligand derived from dipicolinic acid and its respective Fe(iii) high-spin complex were synthesized to study their electrocatalytic HER performance. Our investigation highlights that through electro-oxidation reactions, the *p*-chloro-substituted pyridine rings in the ligand structure can be utilized to immobilize the molecular complexes onto carbon surfaces. Given the wide range of similar molecular complexes available, their heterogenization through electrodeposition *via* electro-oxidation reactions is often an interesting area to explore. It should be noted that this work was intended to highlight the potential of substituted pyridine-2,6-disubstituted metal complexes in the development of economically viable heterogenous dihydrogen production systems. The excellent stability showcased by these modified electrodes makes their utilization in electrolyzers for seawater splitting possible, and corresponding studies are underway.

## Author contributions

BM contributed to conceptualization, data curation, formal analysis, investigation, methodology, validation and writing-original draft. SS contributed to formal analysis, methodology and writing-review and editing. BPM contributed to data curation, software and writing-original draft. MG contributed to conceptualization, funding acquisition, project administration, supervision, validation and writing-review and editing.

## Conflicts of interest

There are no conflicts to declare.

## Supplementary Material

RA-016-D5RA09721E-s001

RA-016-D5RA09721E-s002

## Data Availability

CCDC 2506039 contains the supplementary crystallographic data for this paper.^29^ The data supporting this article have been included as part of the supplementary information (SI). Supplementary information: crystallographic information, additional CV data and mechanistic information for electrochemical immobilization of complex 1. See DOI: https://doi.org/10.1039/d5ra09721e.
