# Supplementary material for: An iron-based molecular cathode for alkaline electrocatalytic hydrogen evolution
Source: RSC Adv. 2026 Apr 20;16(22):20427–32. doi: 10.1039/d5ra09721e (PMC13093890; doi:10.1039/d5ra09721e)
Supplement: RA-016-D5RA09721E-s001 [file RA-016-D5RA09721E-s001.pdf]

Supplementary Information (SI) for RSC Advances.
This journal is © The Royal Society of Chemistry 2026

Iron based Molecular Cathode for Alkaline Electrocatalytic Hydrogen Evolution

Bharath M,[a] Srijit Sen,[a] Bhupendra P Mali [a] and Munmun Ghosh*[a]

[a] Department of chemistry, Ashoka University, Rajiv Gandhi Education city, Haryana-131029, India

Email: *munmun.ghosh@ashoka.edu.in

Table of Contents

Contents	Page Number
1. Synthesis of ligand and complex	4
2. Characterization of ligand	5
3. Characterization of complex	6-8
4. Electrochemical analysis	9-14
5. Post catalytic analysis	15
6. References	15

S1. Synthesis of ligand and complex 1

Synthesis of 4-chloropyridine-2,6-dicarboxylic acid: 3g of chelidamic acid (**a**) was added to 10 ml of $SOCl_2$ with catalytic amount of DMF and the suspension was refluxed for 24 h to yield b. The greenish yellow solution obtained was vacuum dried (**b**) and 12 ml methanol was added under cooling condition and the system was stirred for 1h. Later excess methanol was removed under reduced pressure, the residue was dissolved in $CHCl_3$ and washed with H_2O (2 times) , brine (2 times). The organic phase was dried with Na_2SO_4, filtered and the excess solvent was removed under reduced pressure to yield 4-chloropyridine-2,6-dicarboxylic acid monomethyl ester (c). ^{1}H NMR spectrum of **c** (400 MHz, DMSO-d_6): δ = 8.3 (s, 2H, py), 4.0 (s, 2H), 7.95 (s, 2H).

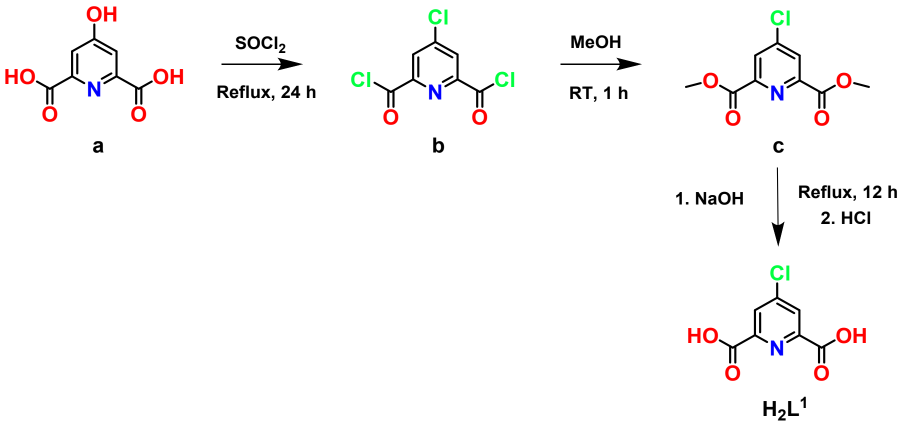

Scheme S1. Synthesis of H_2L^1

10ml 2.5 M NaOH solution was added to c and the mixture was refluxed for 6h. The reaction mixture was allowed to cool down to room temperature. The suspension was filtered, and the pH of the solution was adjusted to 2 by slowly adding 10% HCl solution. The white solid (H_2L^1) product obtained was filtered and washed with cold water several times.

Complex **1** was synthesized by reacting an aqueous solution of iron (III) nitrate nonahydrate with methanolic solution of 4-chloro pyridine-2,6-dicarboxylic acid and pyridine at 100°C.[1-3] Crude orange product was obtained after slow evaporation of reaction mixture which used for further characterization and electrochemical studies. Elemental analysis for **1** ($C_{22}H_{21}FeN_5O_{15}$), Found (%): C- 42.65, H- 1.90, N- 8.24 ; Calculated (%): C- 42.65, H- 1.88, N- 7.85. HRMS for **1** ($C_{14}H_4Cl_2FeN_2O_8$): m/z = 453.87 (calculated), 453.86 (observed). Selected Infrared (IR) spectral data (cm^{-1}) : 3073 (sh, N-H) and 1647 (s, C=O) .

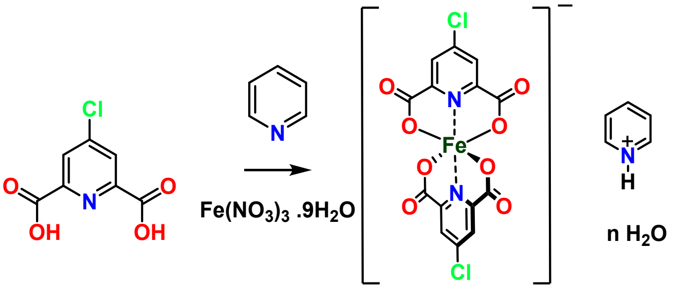

Scheme S2. Synthesis of complex **1**

S2. Characterization of ligand

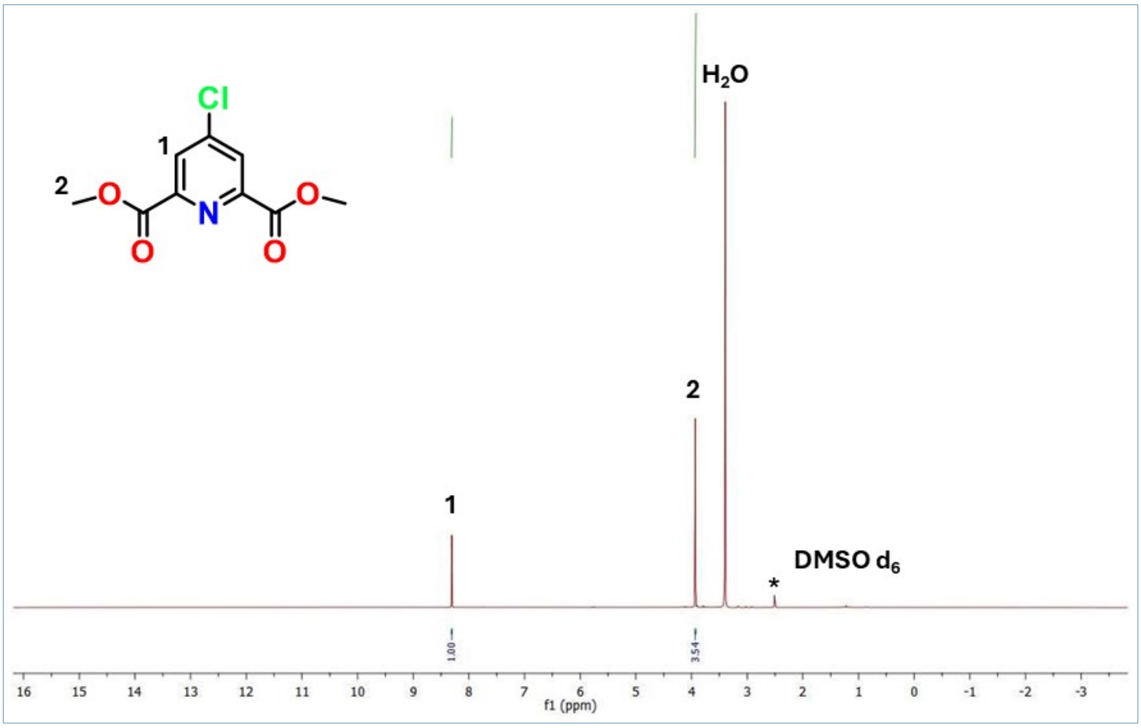

Figure S1. ^{1}H NMR spectrum of **c** in DMSO-d_6 (400 MHz)

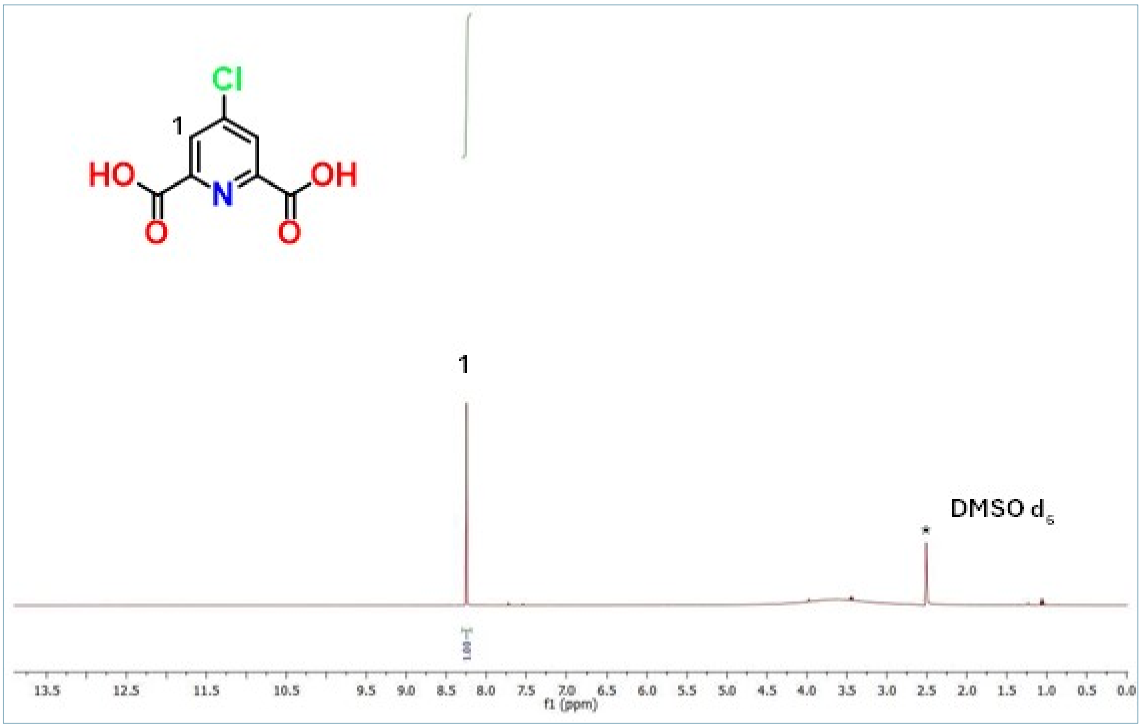

Figure S2. ^{1}H NMR spectrum of ligand H_2L^1 in DMSO-d_6 (400 MHz)

S3. Characterization of metal complex 1

Single crystal XRD structure analysis

Upon successful synthesis of H_2L^1 ligand, single crystals of complex **1** suitable for single-crystal X-ray diffraction (SCXRD) studies were obtained. Single crystal data were collected using a Oxford XCalibur CCD diffractometer equipped with a graphite monochromatic Mo-Kα radiation (λ = 0.71073 Å).The structure was solved using the SHELX program implemented in CrysAllis Pro.[4] All non-hydrogen atoms were refined with anisotropic displacement parameters. All hydrogen atoms were placed in ideal positions and refined using the riding model with appropriate HFIX commands. The diffraction data were collected at room temperature, which resulted in moderately increased atomic displacement parameters across the structure. One chlorine atom in the main molecule exhibited an elongated ellipsoid and residual electron-density features indicative of positional disorder. This atom was therefore modelled over two positions (PART 1 / PART 2) with refined occupancies of approximately 80% and 20%, respectively, without restraints to maintain chemically reasonable geometry and displacement parameters for the disordered components. The PLAT220 alert arises from the larger Ueq(max)/Ueq(min) ratio of the disordered chlorine atom, which is expected for a heavy atom refining over two partially occupied positions at room temperature. The PLAT242 alert reflects a slightly lower Ueq value for atom C18 relative to its neighbours. Inspection of electron-density maps shows no alternative disorder at this position and the bond distances around C18 remain chemically reasonable; the alert is attributed to local refinement effects and the limited high-angle data inherent to room-temperature measurements. No chemically meaningful alternative disorder model was found, and the current refinement provides the best representation of the structure. The Fourier map also showed disordered methanol molecules which are highly disordered couldn't modelled sensible, the PLATON Squeeze analysis revealed 26 electrons per unit cell equivalent to 1.4 methanol molecules per unit cell. The final R values and residual electron-density peaks are within acceptable limits for a room-temperature dataset involving heavy atoms and minor positional disorder.

CCDC number: **2506039**

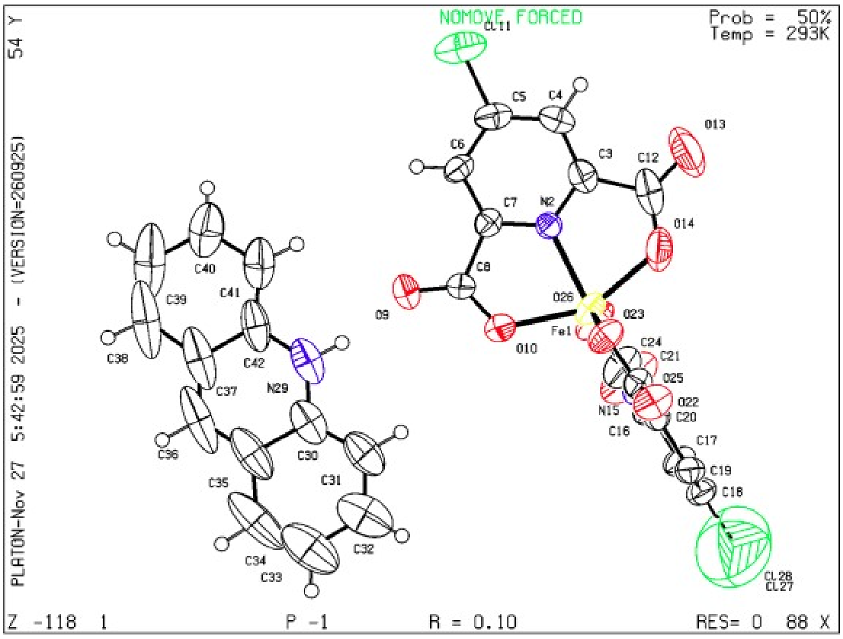

Figure S3. ORTEP diagram of **1** at 50% probability. Colour code: carbon- grey; iron-yellow; nitrogen-blue; and oxygen-red.

Table S1. Crystal data and structure refinement for **1**.

Identification code	SHELX	
Empirical formula	C27 H13 Fe N3 O8 Cl2	
Temperature	293 K	
Wavelength	0.71073 Å	
Crystal system	Triclinic	
Space group	P-1	
Unit cell dimensions	a = 7.8856(4) Å	α= 64.423(6)°.
	b = 12.9940(5) Å	β= 75.382(3)°.
	c = 14.5983(7) Å	γ = 87.511(5)°.
Volume	1301.88(12) Å^3	
Z	3	
F(000)	640.0	
Reflections collected	8106	

High resolution mass spectrometry analysis

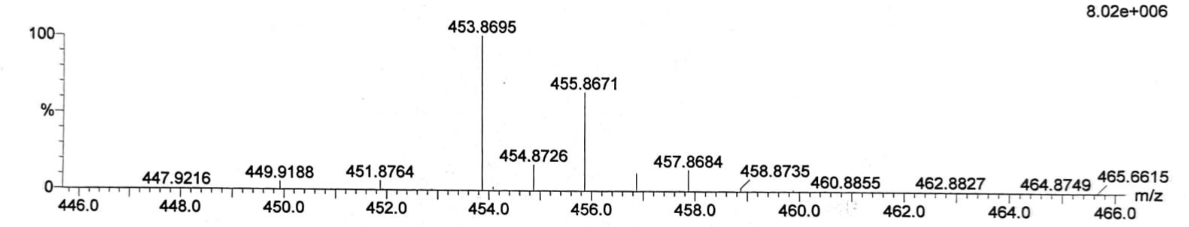

Mass	Calc. Mass	mDa	PPM	DBE	i-FIT	Norm	Conf(%)	Formula
Minimum:				-1.5				
Maximum:		5.0	5.0	50.0				
453.8695	453.8696	-0.1	-0.2	2.0	865.8	38.266	0.00	C8 H13 N2 O7 Na Cl2 Fe2
	453.8694	0.1	0.2	13.0	827.5	0.000	100.00	C14 H4 N2 O8 Cl2 Fe
	453.8694	0.1	0.2	0.5	865.8	38.267	0.00	C7 H14 N O10 Cl2 Fe2
	453.8697	-0.2	-0.4	14.5	865.8	38.264	0.00	C14 H4 N3 O8 Fe2
	453.8691	0.4	0.9	6.5	865.8	38.265	0.00	C11 H10 N O8 Na Cl Fe2
	453.8702	-0.7	-1.5	10.0	865.8	38.265	0.00	C11 H7 N4 O7 Cl Fe2
	453.8687	0.8	1.8	11.0	865.8	38.264	0.00	C14 H7 O9 Na Fe2
	453.8680	1.5	3.3	1.0	865.8	38.267	0.00	C5 H12 N4 O9 Cl2 Fe2
	453.8678	1.7	3.7	7.0	865.8	38.266	0.00	C9 H8 N4 O7 Na Cl Fe2
	453.8675	2.0	4.4	5.5	865.8	38.266	0.00	C8 H9 N3 O10 Cl Fe2

Figure S4. ESI mass spectrum of complex **1** recorded positive ion mode.$C_{14}H_4Cl_2FeN_2O_8$: [M] = 453.87(Calculated), 453.8695(observed).

X-ray photoelectron spectroscopy analysis

Powder samples of complex **1** dried under vacuum were used to analyze the XPS. The binding energies of all spectra were corrected by using the difference between the observed C 1s peak energy and the peak energy of adventitious carbon (284.5 eV). Spectra were fit with a Shirley type of background using CASAXPS version 2.3.19PR1.0 software.

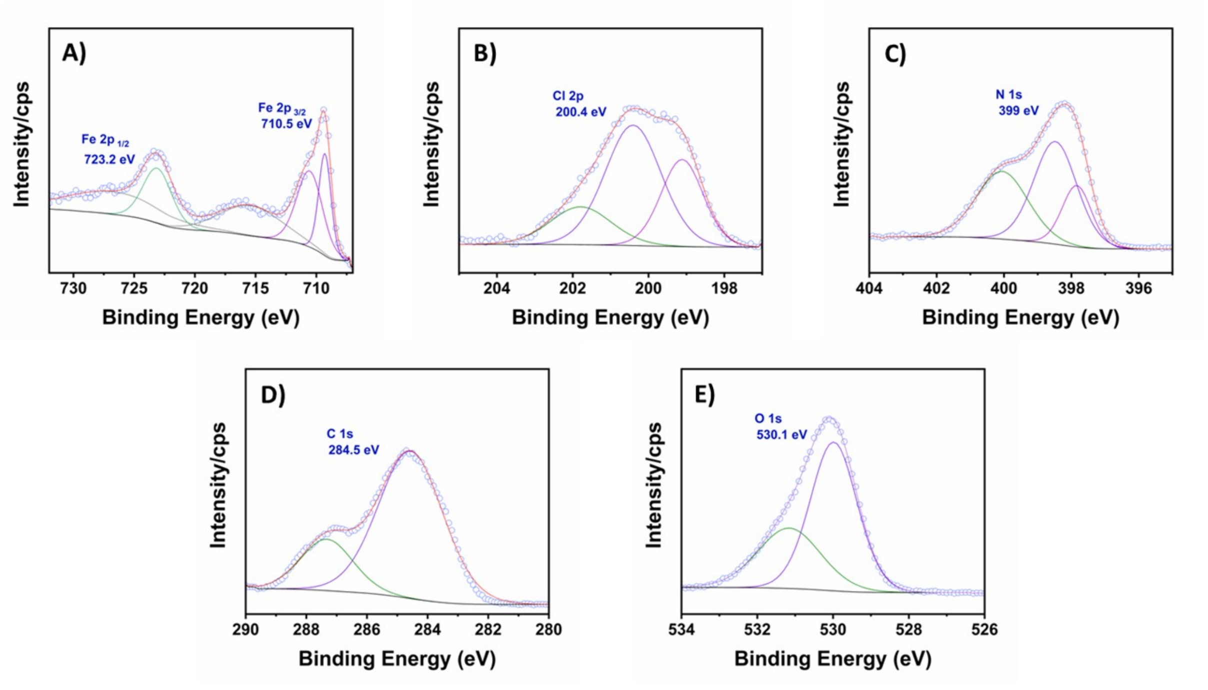

Figure S5. High resolution XPS spectra for elements (A) Fe 2p, (B) Cl 2p, (C) N 1s, (D) C 1s and (E) O 1s of complex **1**.

S4. Electrochemical analysis

All electrochemical studies were conducted at ambient temperatures using an Autolab PGSTAT potentiostat. Cyclic voltammetry analysis was done in a standard three-electrode cell employing a carbon fibre paper (CFP) electrode as the working electrode (WE), $Ag/AgNO_3$ (non-aqueous reactions)or Ag/AgCl (aqueous reactions) as reference electrode (RE) and a spiral platinum wire as counter electrode (CE). For electrochemical deposition of catalyst, 0.6 mM catalyst solutions were prepared in 0.1 M $TBABF_4$/DMF which are purged with nitrogen for 20 minutes. Then CFP electrodes were cycled between potentials -0.2 to 1.2 for several cycles (2 cycles, 5 cycles, 10 cycles and 20 cycles respectively) in a three electrode cell setup. Electrodes procured after cyclic voltammetry analysis were washed thoroughly with DMF several times to eliminate any loosely bound catalyst.

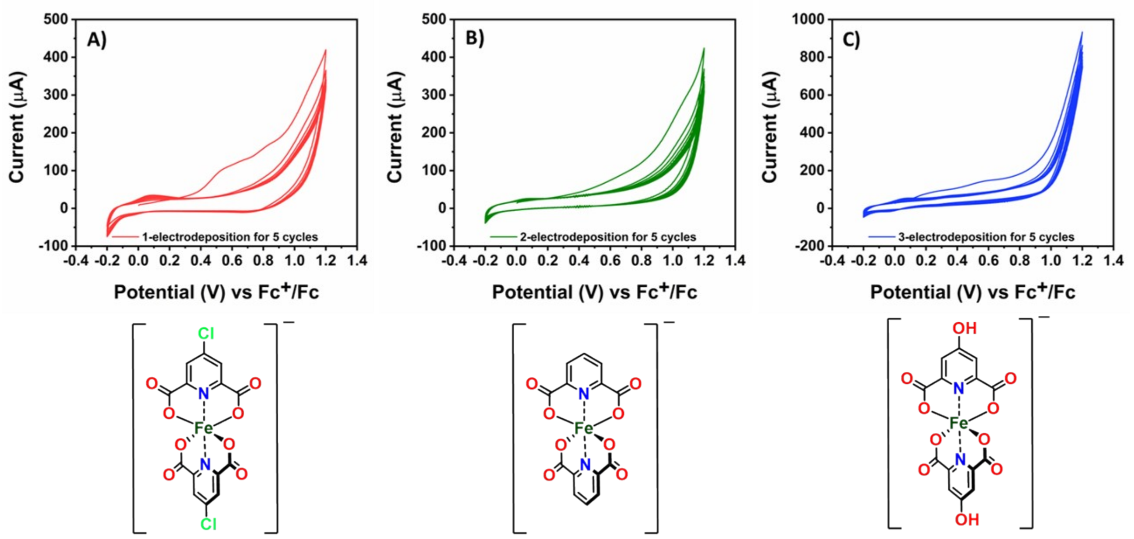

Complex 1 Complex 2 Complex 3

Figure S6. A) Electrochemical deposition of complex **1** on CFP through cyclic voltammetry for 5 continuous scans at scan rate 100 mV/s in 0.1 M $TBABF_4$/DMF under nitrogen saturated conditions. B and C) Electrochemical deposition of control catalyst complex **2** and **3** on CFP through cyclic voltammetry for 5 continuous scans at scan rate 100 mV/s in 0.1 M $TBABF_4$/DMF under nitrogen saturated conditions. (Complex **2** and **3** were synthesized following reported procedures)[1]

For the hydrogen evolution reaction, a three-electrode setup was used, with the catalyst loaded CFP as the working electrode, Ag/AgCl (3M KCl) and a large surface area platinum wire was used as the reference and counter electrode respectively. As electrolyte, 1M KOH solution was prepared from KOH pellets (99.99% semiconductor grade, Sigma-Aldrich). Potentials in aqueous solutions were converted to the RHE scale following the standard conversion from the potential of an Ag/AgCl (3M KCl) reference electrode. Potentials used in this work refer to the reversible hydrogen reference electrode (RHE) scale and the conversion from the Ag/AgCl (3 M KCl) to the RHE scale, are done using the following expression:

$$E_{RHE} = E_{app} + E^0_{Ag/AgCl} + 0.059 \times pH$$

where E_{RHE} is the electrode potential in the RHE scale (in V); E_{app} is the applied potential versus Ag/AgCl (3 M KCl); E^0Ag/AgCl is the standard potential of the Ag/AgCl (3 M KCl) reference electrode (0.21 V), and the pH of the 1 M KOH is 13.9.

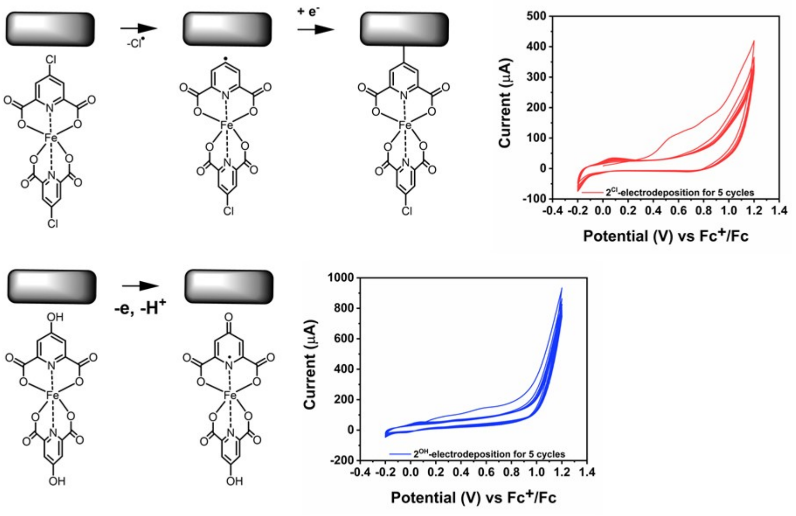

Scheme S3. Proposed mechanism for immobilization of complex **1** on carbon surfaces. A reaction similar to complex **1** is expected in complex **2**, but Cl being a better leaving group made the electrodeposition of complex **1** better than complex **2**. In case of complex **3** due to formation pyridinone species after oxidation immobilization was not possible.

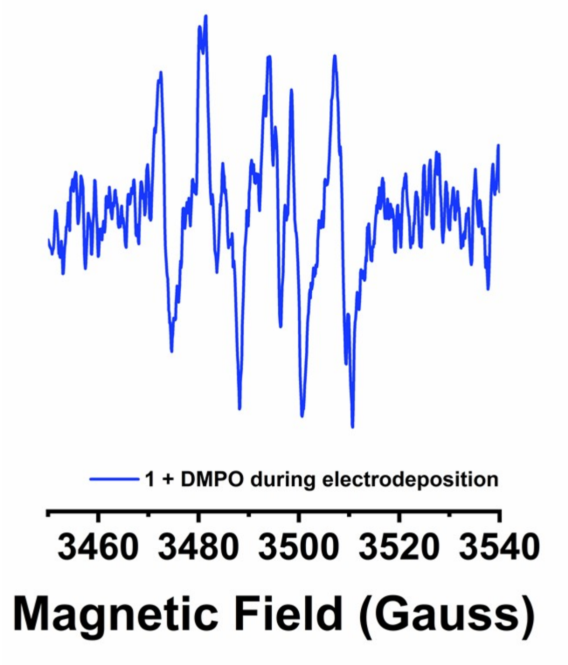

Figure S7. Electron paramagnetic spectra collected at 298 K of DMPO-Cl$^{\cdot}$ adduct showing the hyper fine splitting pattern.

Electrochemical deposition reaction has been performed using dry acetonitrile under inert condition to avoid the interaction of DMPO with water and oxygen.[5, 6]

Electrocatalytic HER performance of catalyst loaded electrodes

Among the modified electrodes, catalyst **1** electrodes were only capable of showing a catalytic response better than the bare CFP electrode. An oxidation event was observed in the case of complex **1** at 0.55V and on successive cycles (5 cycles) the current response for this event significantly reduced indicating the masking of active surface area of the working electrode. Previous reports on electrochemical oxidation of *p*-chloroaniline have shown that the anodic peak of *p*-chloroaniline lies in the potential range of complex **1**. The anodic peak corresponding to *p*-chloroaniline is for the oxidation of chloride ion.[7, 8] We propose that a similar chloride oxidation event is happening in case complex **1**, resulting its electrodeposition on CFP.

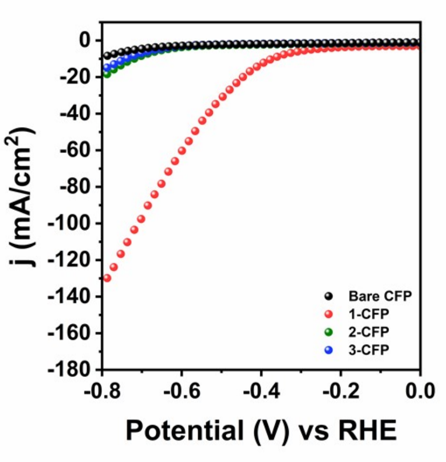

Figure S8. LSV curves for **1-CFP**, **2-CFP** and **3-CFP** electrodes at scan rate 5mV/s at room temperature (without *iR* compensation).

Electrodes procured after electrodeposition were washed thoroughly with DMF several times to eliminate any loosely bound catalyst. These electrodes were then placed in fresh 0.1 M $TBABF_4$/DMF solution to check the presence of catalyst on electrode surface through electrochemical analysis. Redox waves were observed when the modified carbon electrodes (catalyst loaded) were evaluated using cyclic voltammetry analysis. These were compared with bare carbon electrodes for more clarity.

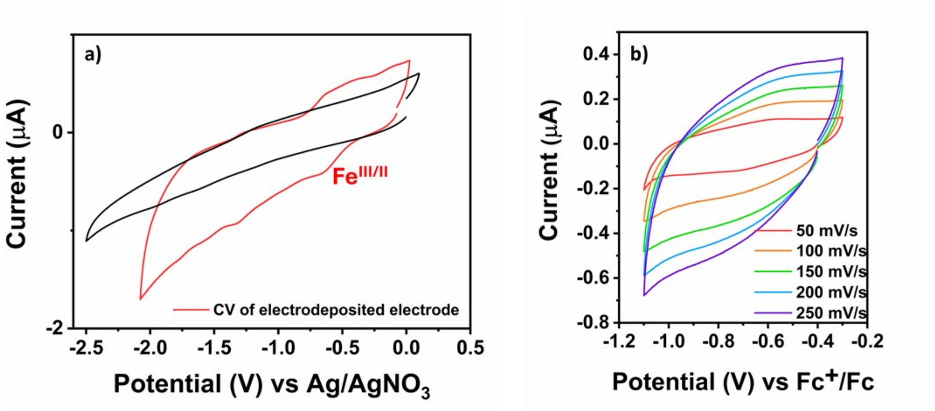

Figure S9. a)Cyclic voltammograms recorded for catalyst **1** loaded carbon electrodes in 0.1M $TBABF_4$/DMF at nitrogen saturated conditions. Black trace denotes the cyclic voltammogram of blank solution with just carbon electrode (No catalyst loaded) and b) variable scan rate analysis for the redox behaviour

Powder X-ray diffraction analysis

To determine the interplanar distance of graphene layers in carbon fiber paper XRD analysis is performed. Using Bragg's law the interplanar distance "d" is calculated.

Bragg's law:

$$n\lambda = 2dsin\theta$$

where n is the order of refection (usually 1), λ is the X-ray wavelength (for Cu K_α, λ = 0.15406 nm) and θ is the angle.

$$d = \frac{\lambda}{2sin\theta}$$

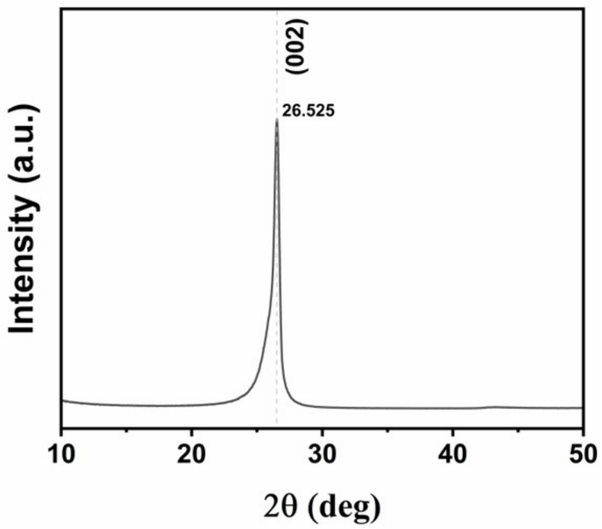

Figure S10. Powder XRD pattern for bare CFP electrode

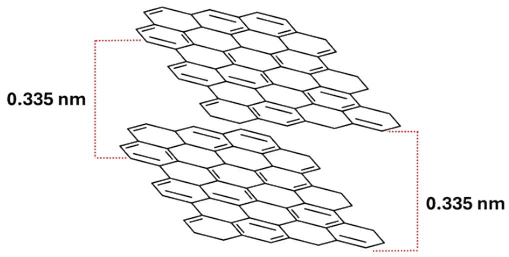

For (002) peak, the corresponding 2θ value is 26.525°, then θ = 13.2625°

$$d = \frac{0.15406}{2\sin(13.2625°)} \approx 0.335\ nm$$

From the calculated interplanar distance between each graphene layer it is evident that the inclusion of these molecular catalyst between these layers are difficult. Thus, the electrochemical deposition of the catalyst **1** should be accounted due to an electrochemical reaction. As we proposed catalyst **1** deposits due to the electro-oxidation reaction and the respective oxidation reaction is the chloride oxidation event (This study also explains why other complexes did not electrodeposit on CFP electrodes).

Optimizing the number of cycles for electrodeposition

For optimization studies 2, 5, 10, 20, 25 cycle electrodeposition reactions were performed. With 5 cycles itself the cyclic voltammograms saturated and further scan resulted in over deposition of catalyst. For easier comparison LSV and FESEM images are provided for electrodes prepared by 5 and 25 CV scans.

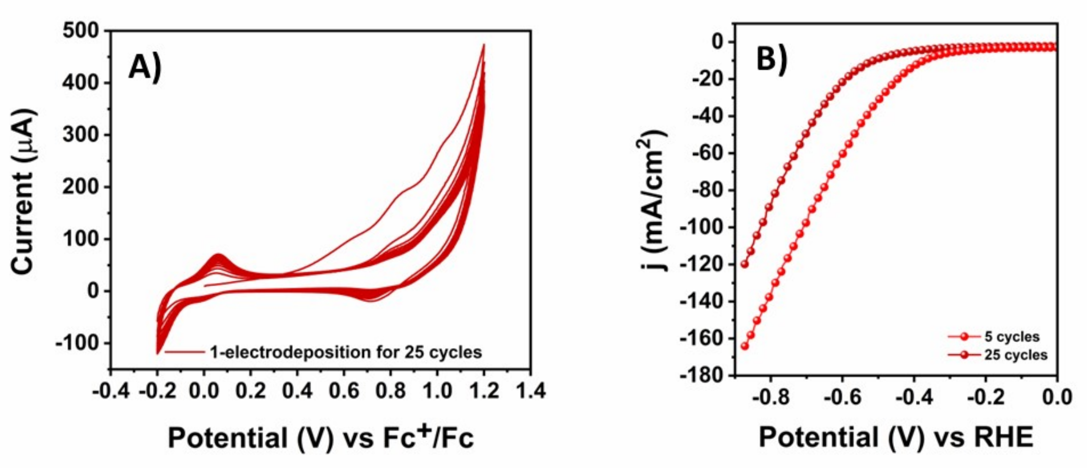

Figure S11. A) Electrochemical deposition of complex **1** on CFP through cyclic voltammetry for 25 continuous scans at scan rate 100 mV/s in 0.1 M $TBABF_4$/DMF under nitrogen saturated conditions. B) LSV curves for **1-CFP** electrodes prepared by 25 cycles and 5cycles electrodeposition (without *iR* compensation).

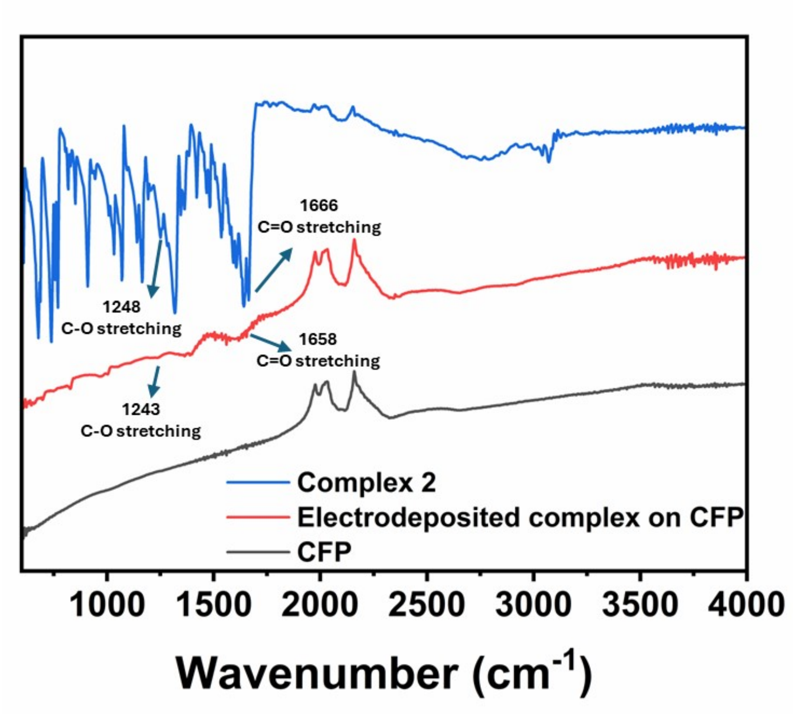

Figure S12. IR analysis performed for CFP electrode before (black) and after catalyst deposition (red). Blue trace shows the IR spectrum of complex **2.**

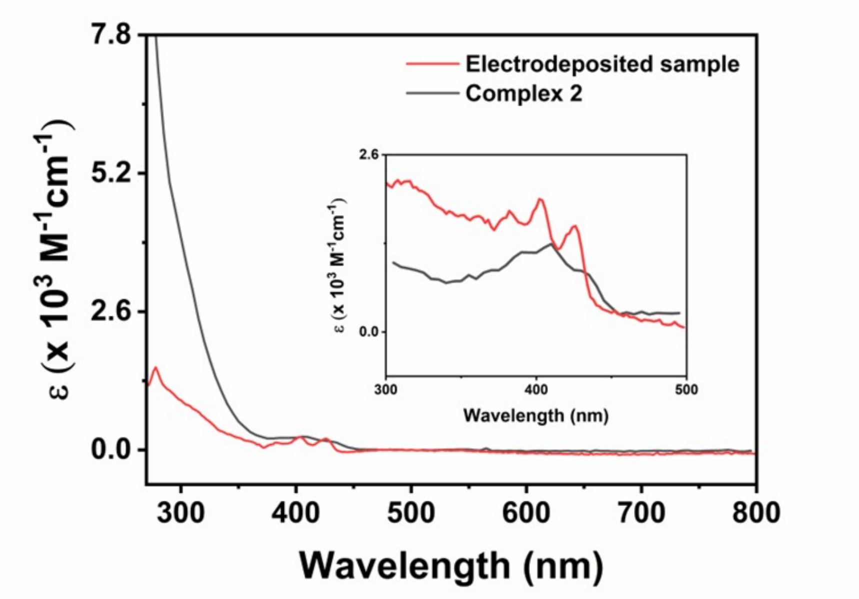

Figure S13. UV-Vis spectra of solution containing electrodeposited species in DMF at room temperature.

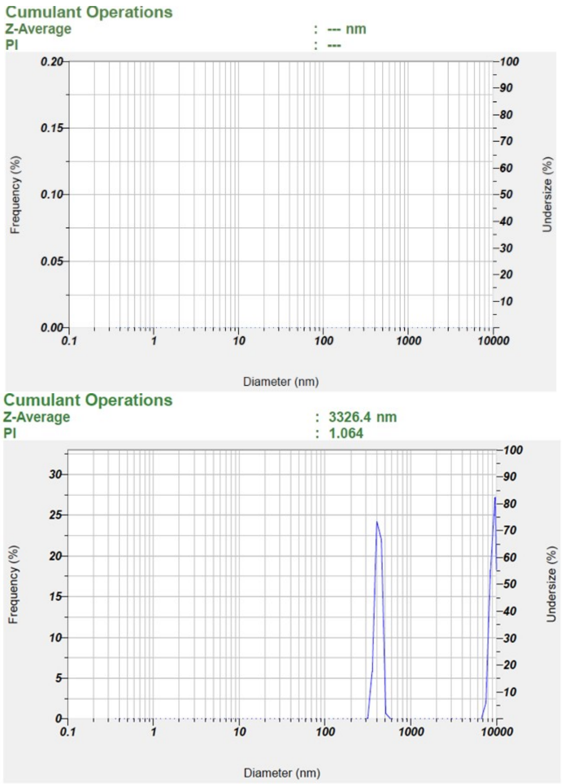

Figure S14. DLS analysis of solution containing electrodeposited species in DMF at room temperature (top). DLS analysis of solution used for electrodeposition (bottom).

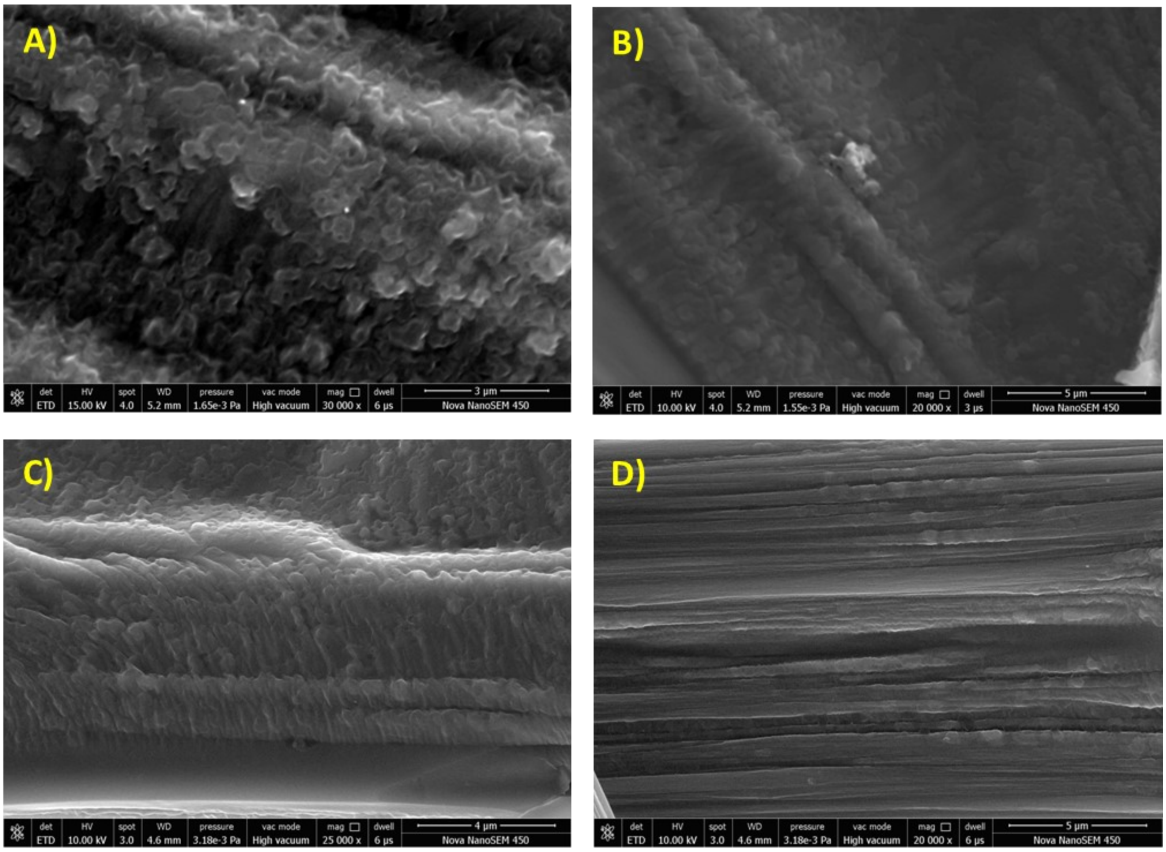

Figure S15. FESEM images of modified electrode **1-CFP** with 25 cycles CV electrodeposition (different magnification A) 3 µm and B) 5µm). FESEM images of modified electrode **1-CFP** with 5 cycles CV electrodeposition (different magnification A) 4 µm and B) 5µm).

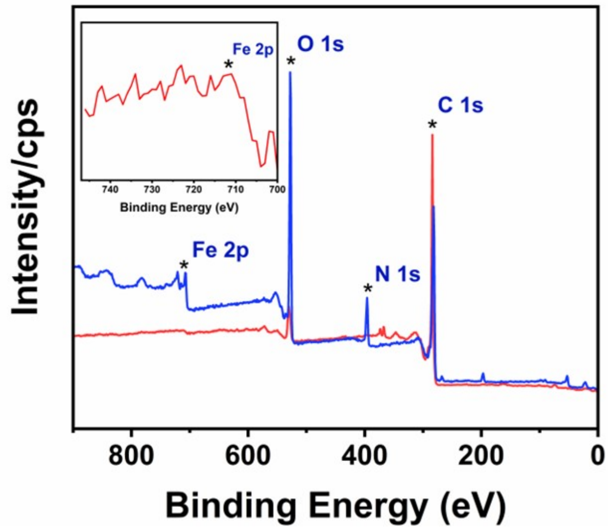

Figure S16. XPS survey spectrum of modified electrode **1-CFP** (red trace) and XPS survey spectrum of complex **1** (blue trace). Inset shows the high resolution spectrum for Fe 2p for **1-CFP**.

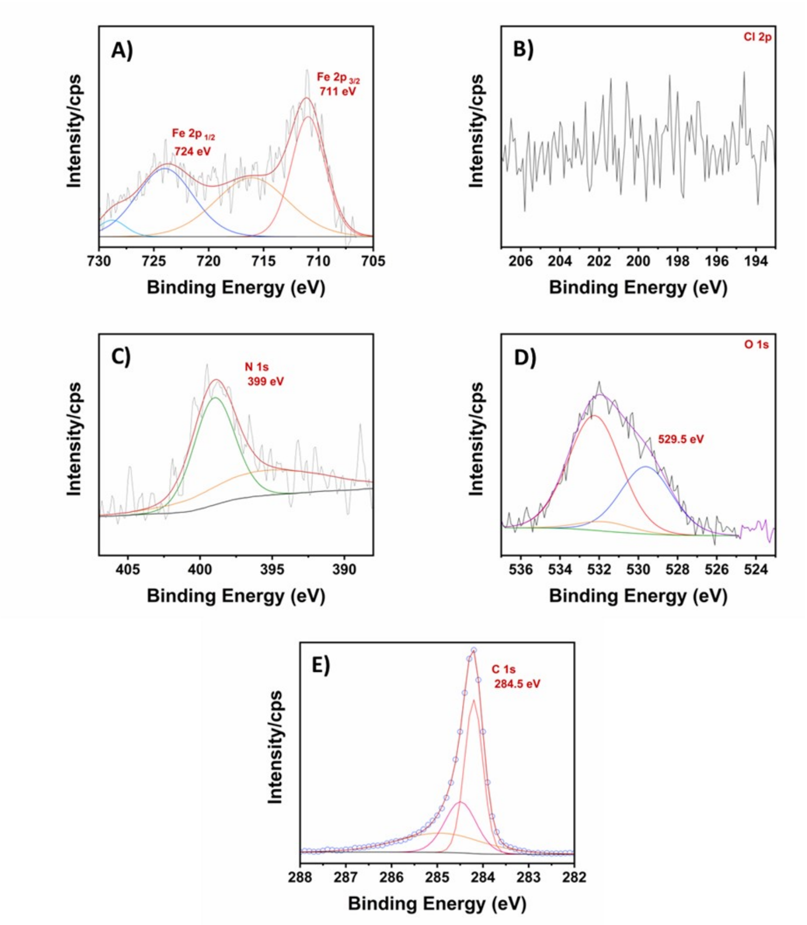

Figure S17. High resolution XPS spectra for elements (A) Fe 2p, (B) Cl 2p, (C) N 1s, (D) O 1s and (E) C 1s of complex **1** loaded electrode **1-CFP**.

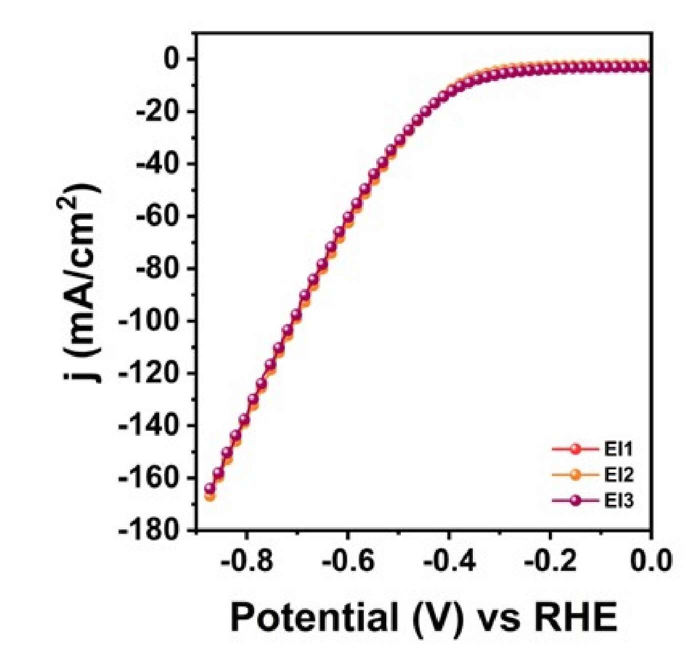

Figure S18. LSV curves for different **1-CFP** electrodes (prepared through 5 cycles electrodeposition) at scan rate 5mV/s at room temperature (without *iR* compensation)

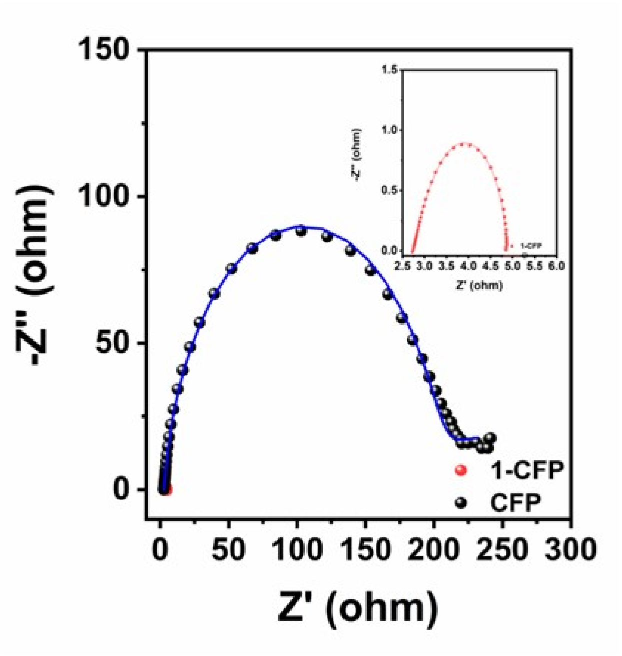

Figure S19. Electrochemical impedance spectra obtained at an overpotential of 400mV for **1-CFP** electrode.

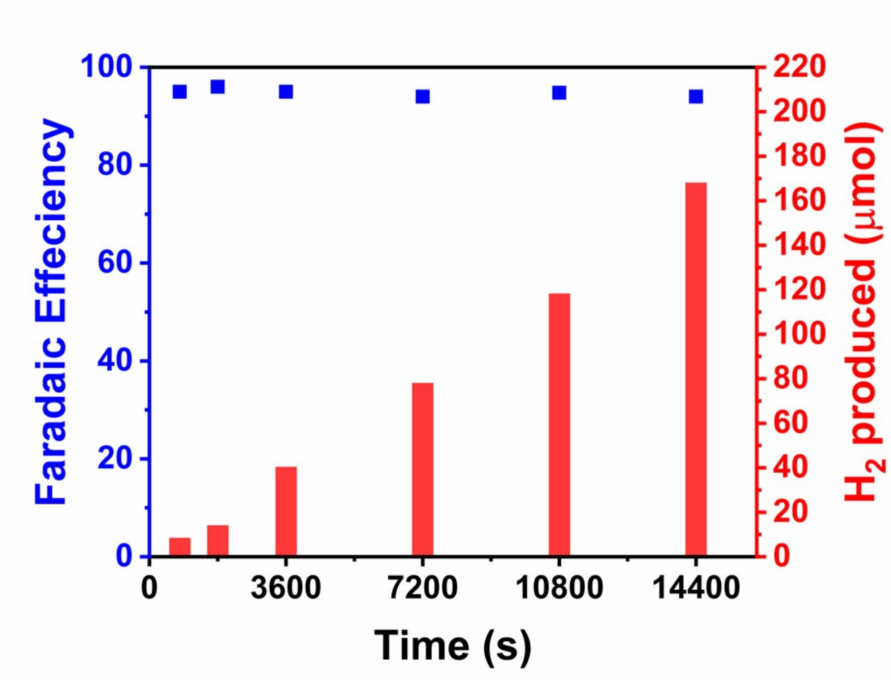

Figure S20. Bulk electrolysis experiment performed for **1-CFP** and respective Faradaic efficiency calculation.

S5. Post catalytic analysis

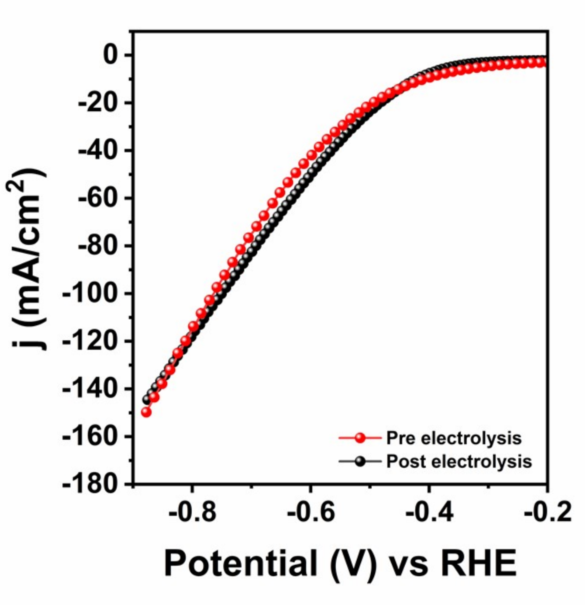

Figure S21. LSV curves recorded for 1-CFP electrodes before and after electrolysis experiment (without iR compensation).

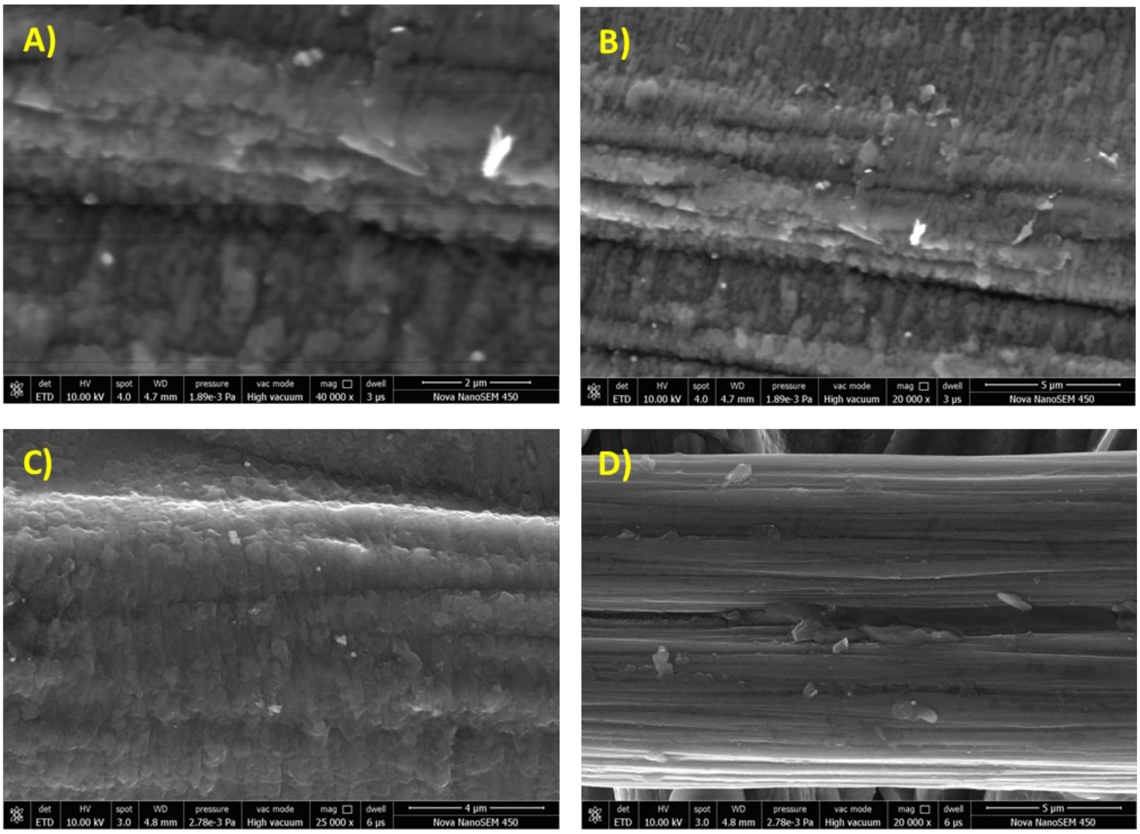

Figure S22. FESEM images of modified electrode **1-CFP** with 25 cycles CV electrodeposition (different magnification A) 2 µm and B) 5µm) after bulk electrolysis. FESEM images of modified electrode **1-CFP** with 5 cycles CV electrodeposition (different magnification A) 4 µm and B) 5µm) after bulk electrolysis.

S6. References

1. Z. Derikvand, A. Nemati, A. Shokrollahi and F. Zarghampour, *Inorganica Chimica Acta*, 2012, **392**, 362-373.
2. M. Mirzaei, H. Eshtiagh-Hosseini, M. Shamsipur, M. Saeedi, M. Ardalani, A. Bauzá, J. T. Mague, A. Frontera and M. Habibi, *RSC Advances*, 2015, **5**, 72923-72936.
3. M. Mahjoobizadeh, M. Mirzaei, A. Bauzá, V. Lippolis, M. C. Aragoni, M. Shamsipur, M. Ghanbari and A. Frontera, *ChemistrySelect*, 2016, **1**, 1556-1566.
4. L. Farrugia, *Journal of Applied Crystallography*, 2012, **45**, 849-854.
5. P. Wang, L. Wang, R. Xiao, S. Qiu, J. Cao, Y. Fu, S. Bai and Z. Wang, *Water Research*, 2025, **285**, 124100.
6. A. Chakraborty, A. Alam, U. Pal, A. Sinha, S. Das, T. Saha-Dasgupta and P. Pachfule, *Nature Communications*, 2025, **16**, 503.
7. C. Amatore, G. Farsang, E. Maisonhaute and P. Simon, *Journal of Electroanalytical Chemistry*, 1999, **462**, 55-62.
8. M. Kádár, Z. Nagy, T. Karancsi and G. Farsang, *Electrochimica Acta*, 2001, **46**, 1297-1306.
